# The Spanish translation, adaptation, and validation of a Community-Engaged Research survey and a pragmatic short version: Encuesta Comunitaria and FUERTES

**DOI:** 10.1017/cts.2024.613

**Published:** 2024-10-16

**Authors:** Patricia Rodriguez Espinosa, Juan M. Peña, Carlos Devia, Blake Boursaw, Magdalena Avila, Diana Rudametkin, Sergio Aguilar-Gaxiola, Margarita Alegria, Lourdes E. Soto de Laurido, Edna Acosta Pérez, Nina Wallerstein

**Affiliations:** 1 Department of Epidemiology and Population Health, Stanford University School of Medicine, Stanford, CA, USA; 2 Office of Community Engagement, Stanford School of Medicine, Stanford, CA, USA; 3 Department of Psychology, San Jose State University, San Jose, CA, USA; 4 CUNY Graduate School of Public Health and Health Policy, New York, NY, USA; 5 University of New Mexico, Albuquerque, NM, USA; 6 University of California, Medical Interpreting Services, Davis, USA; 7 University of California, Davis, USA; 8 Massachusetts General Hospital, Boston, MA, USA; 9 Harvard Medical School, Boston, MA, USA; 10 University of Puerto Rico, San Juan, Puerto Rico; 11 Center for Participatory Research, University of New Mexico, Albuquerque, NM, USA

**Keywords:** Community-based participatory research, Latino/a/x, Spanish, evaluation tools, Community-Engaged Research, assessment instruments

## Abstract

**Introduction:**

Community-Engaged Research (CEnR) and Community-Based Participatory Research (CBPR) require validated measures and metrics for evaluating research partnerships and outcomes. There is a need to adapt and translate existing measures for practical use with diverse and non-English-speaking communities. This paper describes the Spanish translation and adaptation of Engage for Equity’s Community Engagement Survey (E^2^ CES), a nationally validated and empirically-supported CEnR evaluation tool, into the full-length “*Encuesta Comunitaria*,” and a pragmatic shorter version “*Fortaleciendo y Uniendo EsfueRzos Transdisciplinarios para Equidad de Salud*” (FUERTES).

**Methods:**

Community and academic partners from the mainland US, Puerto Rico, and Nicaragua participated in translating and adapting E^2^ CES, preserving content validity, psychometric properties, and importance to stakeholders of items, scales, and CBPR constructs (contexts, partnership processes, intervention and research actions, and outcomes). Internal consistency was assessed using Cronbach’s alpha and convergent validity was assessed via a correlation matrix among scales.

**Results:**

*Encuesta Comunitaria* respondents (*N* = 57) self-identified as primarily Latinos/as/x (97%), female (74%), and academics (61%). Cronbach’s alpha values ranged from 0.72 to 0.88 for items in the context domain to 0.90–0.92 for items in the intervention/research domain. Correlations were found as expected among subscales, with the strongest relationships found for subscales within the same CBPR domain. Results informed the creation of FUERTES.

**Conclusions:**

*Encuenta Comunitaria* and FUERTES offer CEnR/CBPR practitioners two validated instruments for assessing their research partnering practices, and outcomes. Moreover, FUERTES meets the need for shorter pragmatic tools. These measures can further strengthen CEnR/CBPR involving Latino/a/x communities within the US, Latin America, and globally.

## Introduction

Community-Engaged Research (CEnR) and Community-Based Participatory Research (CBPR) have gained popularity over the past decades as valued research approaches [1]. Funders and accrediting bodies increasingly require evidence of partnerships between communities and institutions of higher learning and research [2]. Based on empirical frameworks, tools, and resources to guide effective partnering, evaluation, and reflexivity have emerged as best practices to successfully support partnership capacity building, meaningful community engagement, and adherence to CEnR and CBPR principles [3]. Thus, having validated instruments and metrics for evaluating research partnerships and outcomes is crucial. Moreover, such tools should be adapted and translated for pragmatic use with communities from diverse backgrounds and non-English speaking communities.

The National Academy of Medicine (NAM) Organizing Committee on Assessing Meaningful Community Engagement in Health & Health Care Policies & Programs recently released 28 assessment instruments or measures, each providing standard questions or question sets to assess community engagement consistently. These measures are meant to help potential users support the rigorous assessment and evaluation of CEnR and CBPR and map onto the new NAM Assessing Community Engagement Conceptual Model (ACE-CoM) [4]. However, only four were validated with national samples, and even fewer comprehensively covered the domains of the Organizing Committee’s new model [5]. Moreover, few were available in Spanish, which can be critical for partnerships working with the Latino/a/x community, the largest and youngest racial/ethnic minority group in the US [6,7]. Among the three identified Spanish measures in the NAM review, two came from a long-term National Institute of Health-funded research initiative, Engage for Equity [1]. One is the *Encuesta Comunitaria* (or Community Engagement Survey in English), a vital focus of this manuscript. The second is its partner survey, *Encuesta de Informante Clave* (or Key Informant Survey in English), which is directed at principal investigators or project directors to assess the partnership, such as funding source and amount, Institutional Review Board (IRB) training, etc. [8].

One of the four measures identified with national validation and covering most constructs in the NAM ACE-CoM also comes from Engage for Equity, a collaboration between the University of New Mexico Center for Participatory Research (CPR) and national partners [1]. In two successive data collection waves of the internet-based survey tool, Engage for Equity Community Engagement Survey (E^2^ CES) [9,10]. the Engage for Equity team assessed close to 400 partnering teams to identify best-partnering practices associated with outcomes [11,12]. In the second data collection wave, a Spanish Version, *Encuesta Comunitaria*, was also developed and tested for validation. Two shorter versions of the CES and *Encuesta Comunitaria* have also been developed, Partnering for Health Improvement and Research Equity (PHIRE) (Oetzel et al., unpublished data [PHIRE available from nwallerstein@salud.unm.edu]) in English and *Fortaleciendo y Uniendo EsfueRzos Transdisciplinarios para Equidad de Salud* (FUERTES) in Spanish, respectively.

This manuscript provides an overview of the translation and adaptation process of the long and short Spanish versions of Engage for Equity’s *Encuesta Comunitaria* and FUERTES, respectively. We also provide measurement properties for the *Encuesta Comunitaria*. Both tools can assess CEnR partnering processes, practices, and outcomes within Spanish-speaking communities in the US and Latin America.

## Materials and methods

This study received approval from two IRBs at the University of New Mexico. The first IRB (Protocol #16–098) covered the refinement, Spanish translation, and testing of the E^2^ CES in Puerto Rico and Nicaragua. The second IRB (Protocol # 19–376) covered the quantitative and qualitative process to shorten and translate the E^2^ CES.

### Overview of original English measure: Engage for Equity Community Engagement Survey (E^2^ CES)

E^2^ CES is a valid and reliable tool appropriate for measuring constructs to evaluate CBPR and CEnR partnerships and to apply results for strengthening project processes and outcomes [1,13]. The most recent version of the E^2^ CES measurement instrument includes 81 scale items organized across 23 distinct areas within the four domains of the empirical CBPR model, including context, partnership processes, intervention and research actions, and outcomes [14].

The E^2^ CES was tested in two iterations, in 2015 with 200 partnerships [9] and in 2020 with 179 partnerships [10]. Scales and constructs in the E^2^ CES have demonstrated strong factorial validity, convergent validity, and internal consistency [9,10]. These findings suggest that the measures of the E^2^ CES can be used by CEnR/CBPR practitioners and researchers to evaluate CEnR/CBPR partnerships and to advance the science of CEnR/CBPR. See the UNM CPR website E^2^ tools and resources for the specific items and response options in the Spanish version of the Community Engagement Survey (CPR, https://hsc.unm.edu/population-health/research-centers/center-participatory-research/cbpr-community-engagement/, 2023) and the Engage for Equity website (https://engageforequity.org).

### Linguistic and Pragmatic Adaptation of Engage for Equity’s Community Engagement Survey

As CEnR increases in popularity in many partnerships involving non-English speaking community partners within the US and globally, there is a need for tools that are available in other languages. CBPR has strong roots in Latin America, grounded in Freirean dialogical education and dating back to the first participatory action research conference held in Cartagena, Colombia in 1977 [15,16], and where many Spanish-speaking countries have long histories of community participation, including on projects with U.S. institutions [17]. Spanish-speaking partnerships have been of particular interest, with data from the NIH RePORTER showing that from 2020 through 2024, the number of CEnR or CBPR federal grants involving Spanish-speaking participants increased from 23 on or before December 2020 to 274 in May 2024. However, to our knowledge, no study has developed validated measures for Spanish-speaking partnerships.

**Figure 1.**
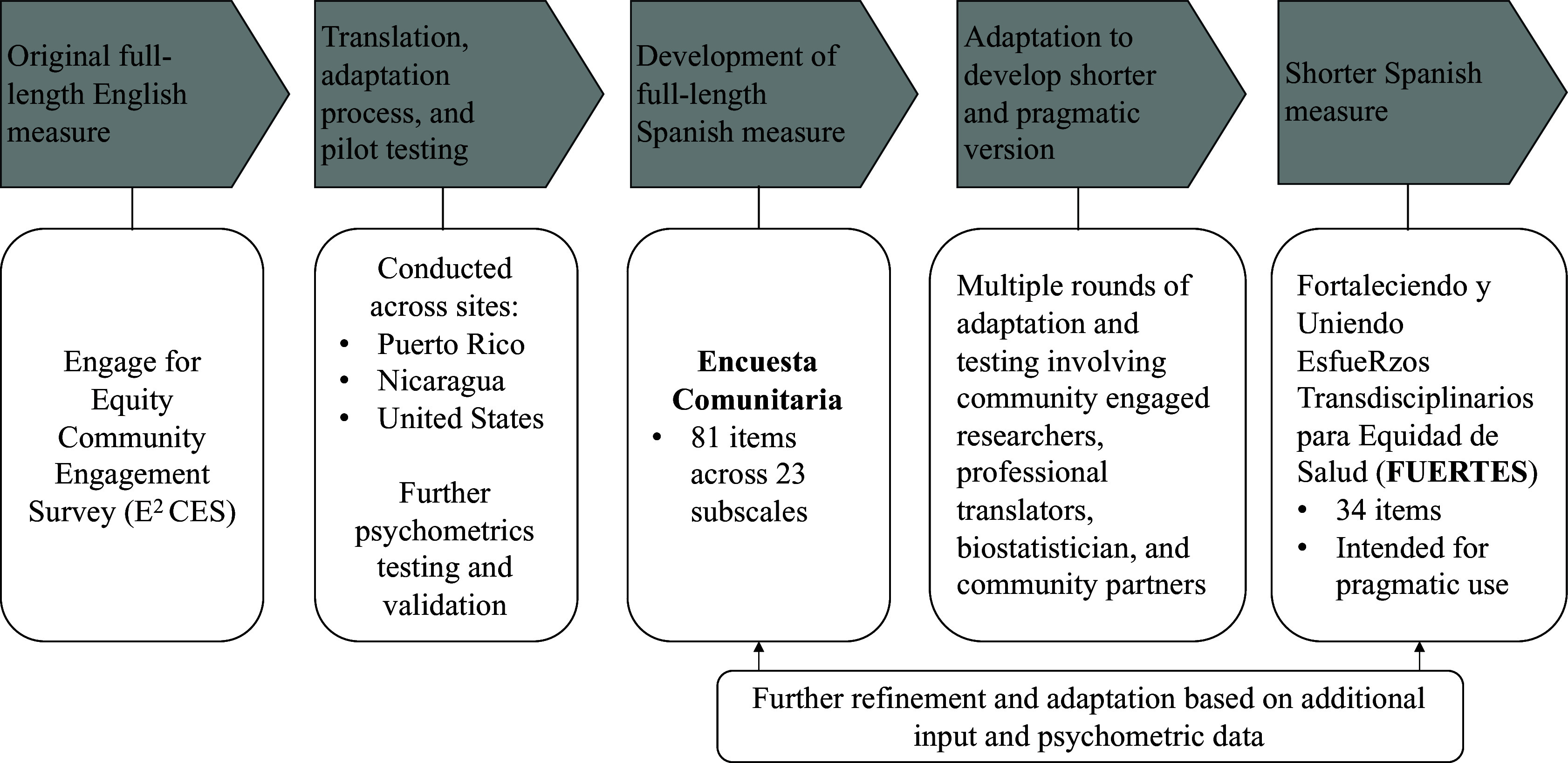
Overview of measures and process for translation, adaptation, and refinement.

While the E^2^ CES is an advance in partnership measures, this long form, has a high response burden due to the large number of items, making it difficult for partnerships to incorporate into their regular evaluations of their partnering processes and adherence to best practices for CEnR. According to Glasgow and Riley (2013), pragmatic measures should, at minimum, be necessary to stakeholders, have a low burden, be actionable, and be sensitive to change [18]. To develop shorter pragmatic measures of CBPR processes and outcomes, we employed a method to preserve content validity, psychometric properties, and importance to stakeholders of items, scales, and constructs from the original E^2^ CES [18]. This process is described in detail below (see Figure 1).

### Overall Spanish translation and cultural adaptation processes

Although Spanish-speaking Latino/a/x populations are considered one ethnic group in the US, they represent a heterogeneous mix of cultures from more than 20 countries in Latin America and the Caribbean [6,19]. The E^2^ CES was translated and adapted from English to a Spanish version, called *Encuesta Comunitaria,* that could be understood by diverse Spanish speakers in the US (e.g., from Mexico, Cuba, Puerto Rico, etc.) and that would serve as a basis for adaptations to specific cultural contexts. This full-length Spanish version mirrored the multiple scales found in the English E^2^ CES, which included validated measures across the four domains of the CBPR model, [20] the development of new scales [10] informed by prior studies, [9] and consultation with a national advisory team of community and academic researchers [1,13].

The translation and adaptation processes were carried out in collaboration with academic experts in social, clinical, and community research based in the mainland US and Puerto Rico. To promote maximum language and cultural equivalency of the measures for the study, independent bilingual CBPR researchers undertook the following recommended published steps [21–23]: (1) the original version was translated into Spanish by a professional, certified translator independent from the original measure development and study team; (2) the Puerto Rican team made some revisions comparing this Spanish translation item-by-item to the original E^2^ CES English version in ∼12 hours of meetings, including discussing discrepancies and suggestions; (3) the items were back-translated to compare the wording with the original version and to re-formulate those items which did not keep their original meaning; and (4) cognitive debriefing interviews were conducted by Puerto Rican partners with five community participants to identify difficulties in comprehension, validating the translation and adaptation process.

This refined Spanish version was also reviewed by bilingual Engage for Equity team members (PRE, MA, and LM) who had a deep familiarity with the constructs assessed in the English version. These three bilingual researchers, via roundtable discussions, made additional wording changes, and any discrepancies, comments, or concerns were further discussed with the original English E^2^ CES project Principal Investigator (NW) and the biostatistician on the team. As a result, *Encuesta Comunitaria* is a culturally and linguistically adapted measure incorporating feedback from individuals representing different U.S. and Latin American communities (i.e., North, Central, and South America, and the Caribbean), as well as the largest Spanish-speaking populations in the U.S., including but not limited to Mexico, Puerto Rico, Cuba, and Colombia.

### Testing *La Encuesta Comunitaria* across sites

In Nicaragua, a nonprofit primary care rural system, AMOS Health and Hope [24], decided to use and test the *Encuesta Comunitaria* with their rural community health workers and national training staff to assess and strengthen their partnering practices with each of their village health committees. In one of their semiannual meetings in Managua, the *Encuesta Comunitaria* was administered in a group (*n* = 21) format, with community health workers and staff being able to ask questions as they took the survey. As an additional benefit, the medical director developed a qualitative checklist based on the survey questions to encourage group dialog and co-learning about the value of implementing partnering best practices.

The *Encuesta Comunitaria* measure was also tested in collaboration with community and academic partners in Puerto Rico. Potential participants who were 21 or older and with no cognitive impairment were recruited through convenience sampling. Invitations to participate in the survey were sent to researchers, leaders, and community-based organizations throughout Puerto Rico via emails or physical flyers. Announcements were also available on the Hispanic Alliance for Clinical and Translational Research (Alliance) websites and partnership universities or organizations. The surveys were hand-delivered or mailed to each interested partner organization. In coordination with the Alliance, the Hispanic in Research Capability Endowment delivered a CBPR workshop open to all Puerto Rican community and academic researchers. The thirty-six people enrolled in the workshop completed the *Encuesta Comunitaria* measure.

### *Encuesta Comunitaria* data analyses

Descriptive statistics were performed for all measures with scale and subscale scores across items and sociodemographic variables of respondents. Consistent with the most recent English version of the CES, data analyses were performed for the same items representing the 23 subscales [10]. Internal consistency values were assessed for each scale using Cronbach’s alpha. We performed correlations to examine the relationship between items representing the four CBPR model domains, contexts, partnership processes, intervention and research, and outcomes [14,25]. All analyses were performed using SPSS Version 29.

## Results

Fifty-seven respondents from Puerto Rico (*N* = 36) and Nicaragua (*N* = 21) participated in the study and completed the *Encuesta Comunitaria*. As seen in Table 1, most participants identified as Latinx (97%), female gender (74%), and were academic partners (61%). Under the CBPR conceptual model domains, Cronbach’s alpha values ranged from 0.72 to 0.88 for Context, 0.74–0.93 for Partnership Processes, 0.90–0.92 for Intervention and Research, and 0.45–0.94 for Outcomes. See Table 2 for a complete list of items from scales, subscales, and Cronbach’s alpha across the four domains in the CBPR conceptual model.

**Table 1.** Demographic characteristics of partners completing the Encuesta Comunitaria

Demographic characteristics	Community partners (*N* = 22)	Academic partners (*N* = 35)
Nationality		
• Puerto Rico	15 (68%)	21 (60%)
• Nicaragua	7 (32%)	14 (40%)
Race/ethnicity		
• American Indian/Native American	0 (0%)	1 (3%)
• Black/African American	0 (0%)	1 (3%)
• White	2 (9%)	1 (3%)
• Hispanic/Latinx	22 (100%)	34 (94%)
Gender		
• Female	15 (68%)	27 (77%)
• Male	7 (32%)	8 (23%)

For race/ethnicity, respondents had the option to select all that applied. Therefore, total values may not add up to 100%. No respondents identified as Asian, Native Hawaiian, or Pacific Islander.

**Table 2.** Descriptive statistics for items, subscales, and scales of practices and outcomes in the Encuesta Comunitaria

Scale, subscale, and specific items			Mean (SD)	Cronbach’s alpha and sample size (N = 57)
***Contexto* (Context)**				
***Capacidad* (Capacity)**				
***Contexto Comunitario y de Capacitación*** (**Community history of organizing**)	Overall		4.76 (1.08)	0.75 *N* = 56
	1. La comunidad o comunidades participando en este proyecto tienen un historial de organizar servicios o eventos		4.98 (1.14)	
	2. La comunidad o comunidades que participan en este proyecto tienen un historial de abogar por igualdad social o de salud		4.80 (1.20)	
	3. Trabajando en conjunto, personas en la comunidad o comunidades participando en este proyecto han, anteriormente, influenciado decisiones que afectaban sus comunidades		4.50 (1.58)	
***Capacidad de Colaboración*** (**Partnership capacity**)	Overall		5.20 (0.77)	0.88 *N* = 56
	1. Habilidades y experiencia		5.18 (0.94)	
	2. Diversidad de los miembros		5.11 (0.95)	
	3. Legitimidad y credibilidad en la comunidad		5.38 (0.89)	
	4. Habilidad de congregar a las personas para juntas/actividades		5.09 (0.96)	
	5. Conexiones con personas y grupos interesados		5.23 (0.99)	
***Superar las Diferencias*** (**Bridging differences**)	Overall		5.08 (0.84)	0.72 *N* = 54
	1. Los colegas comunitarios (como pacientes, miembros de la comunidad, u organizaciones) tienen el conocimiento, habilidades, y confianza en sí mismo para interactuar eficazmente con los colegas académicos (como los individuos de las universidades)		5.04 (1.13)	
	2. Los colegas académicos tienen miembros que son de un origen cultural similar al de los colegas comunitarios		4.96 (1.13)	
	3. Los colegas académicos tienen el conocimiento, habilidades, y confianza en si mismo para interactuar eficazmente con los colegas comunitarios		5.24 (0.85)	
***Procesos de colaboración* (Partnership Processes)**				
***Compromiso al empoderamiento colectivo* (Commitment to collective empowerment)**				
***Principios de Participación Comunitaria*** (**Partnering principles**)		Overall	5.46 (0.71)	0.93 *N* = 55
		1. Este proyecto se basa sobre recursos y la fortaleza en la comunidad	5.45 (0.84)	
		2. Este proyecto facilita colaboraciones equitativas en todas las fases de la investigación	5.16 (1.00)	
		3. Este proyecto ayuda a todos los miembros involucrados a crecer y aprender los unos con los otros	5.60 (0.76)	
		4. Este proyecto equilibra investigación y acción social para el mutuo beneficio de todos los colegas	5.47 (0.84)	
		5. Este proyecto enfatiza los factores que son importantes para la comunidad que afectan al bienestar	5.67 (0.61)	
		6. Este proyecto comunica el conocimiento y los resultados a todos los colegas e involucra a todos en el proceso de difusión	5.33 (1.04)	
		7. Este proyecto considera IAP como un proceso y compromiso a largo plazo	5.55 (0.86)	
***Relevancia Comunitaria* (Community fit)**		Overall	5.61 (0.59)	0.74 *N* = 56
		1. Este proyecto es sensible a la historia de la comunidad	5.59 (0.76)	
		2. Este proyecto integra las palabras y el lenguaje de la comunidad	5.59 (0.78)	
		3. Este proyecto conecta con la manera en que las cosas se hacen en la comunidad	5.64 (0.65)	
***Influencia en la Colaboración* (Influence in the partnership)**		Overall	6.14 (1.11)	0.89 *N* = 55
		1. Tengo influencia sobre las decisiones que esta colaboración hace	5.95 (1.35)	
		2. Mi participación influye a la colaboración a ser más responsiva a la comunidad	6.24 (1.04)	
		3. Puedo influir el trabajo en este proyecto	6.24 (1.28)	
***Reflexividad* (Collective reflection)**		Overall	6.17 (1.04)	0.74 *N* = 57
		1. Nuestra colaboración tiene discusiones sobre nuestro papel en promover estrategias que favorecen igualdad social y de salud	6.46 (0.91)	
		2. Nuestra colaboración evalúa mutuamente lo que hemos hecho bien y como podemos mejorar nuestra colaboración	6.39 (1.00)	
		3. Nuestra colaboración reflexiona en cuestiones de poder y privilegio dentro de nuestra colaboración	5.67 (1.77)	
***Relaciones* (Relationships)**				
**Liderazgo (Leadership)**		Overall	5.52 (0.68)	0.89 *N* = 55
		1. Fomenta la participación activa de los colegas académicos y comunitarios en la toma de decisiones	5.42 (0.92)	
		2. Comunica los objetivos del proyecto	5.50 (0.79)	
		3. Fomenta respeto entre colegas	5.69 (0.61)	
		4. Ayuda a los colegas a ser creativos y a ver las cosas de un modo diferente	5.49 (0.79)	
**Diálogo y Escucha (Dialogue and listening)**		Overall	6.44 (0.82)	0.87 *N* = 55
		1. Mostramos actitudes positivas entre nosotros mismos	6.44 (0.90)	
		2. Todos en nuestra colaboración participamos en nuestras juntas	6.29 (1.03)	
		3. Nos escuchamos entre nosotros mismos	6.58 (0.81)	
***Resolución de conflicto* (Conflict resolution)**		1. Cuando suceden conflictos, trabajamos juntos para resolverlos	6.32 (0.86)	0.75 *N* = 56
		2. Incluso cuando no tenemos acuerdo total, llegamos a un tipo de conceso que todos podemos aceptar	6.27 (1.02)	
***Confianza* (Trust)**		Overall	6.39 (0.67)	0.89 *N* = 57
		1. Yo confio en las decisiones que otros hacen sobre asuntos importantes para nuestros proyectos	6.42 (0.68)	
		2. Puedo depender en las personas con quien trabajo en este proyecto	6.35 (0.83)	
		3. Personas en esta colaboración tienen mucha confianza entre si mismos	6.40 (0.70)	
***Intervención e Investigación* (Intervention and Research)**				
***Participación Comunitaria en acción de investigación* (Community Engagement in research action)**				
***Antecedentes y diseño* (Background and design)**		Overall	4.18 (1.31)	0.91 *N* = 47
		1. Integrar entendimientos comunitarios en las preguntas y métodos de investigación	4.68 (1.22)	
		2. Escribir propuestas para fondos	3.70 (1.73)	
		3. Investigar antecedentes	3.91 (1.49)	
		4. Desarrollar procedimientos de muestreo	3.89 (1.71)	
		5. Reclutamiento de participantes	4.70 (1.41)	
***Análisis y diseminación* (Analysis and dissemination)**		Overall	3.83 (1.59)	0.92 *N* = 48
		1. Interpretación de resultados	4.15 (1.68)	
		2. Escribir reportes y artículos para revistas científicas	3.33 (1.78)	
		3. Presentaciones en juntas y conferencias	4.02 (1.68)	
***Acción comunitaria* (Community action)**		Overall	4.23 (1.48)	0.90 *N* = 50
		1. Informar a la comunidad sobre el progreso de la investigación y los resultados	4.58 (1.53)	
		2. Informar legisladores relevantes sobre los resultados	3.66 (1.86)	
		3. Compartir resultados con otras comunidades	4.02 (1.78)	
		4. Producir resultados útiles para acción y beneficio comunitario	4.66 (1.53)	
***Sinergia de la Colaboración* (Synergy)**				
		Overall	5.42 (0.73)	0.92 *N* = 55
		1. Desarrollan metas que son ampliamente entendidas y apoyadas en la colaboración	5.29 (0.96)	
		2. Desarrollan estrategias que son las más probables de funcionar para la comunidad o personas y grupos interesados en su totalidad	5.38 (0.83)	
		3. Reconocen retos y sugieren buenas soluciones	5.44 (0.86)	
		4. Responden a las necesidades y problemas de la comunidad que representan o comunidad en su totalidad	5.42 (0.79)	
		5. Trabajan bien en conjunto como una colaboración	5.56 (0.71)	
***Resultados* (Outcomes)**				
***Cambio de sistema y capacidad* (System and capacity change)**				
***Ventajas Personales* (Personal benefits)**		Overall	4.85 (1.23)	0.81 *N* = 52
		1. Incremento en el uso por otros de su experiencia o servicios	5.13 (1.39)	
		2. Mayor habilidad de adquirir apoyo financiero adicional	4.62 (1.59)	
		3. Mayor habilidad de buscar educación formal o informal	4.81 (1.34)	
***Resultados de Agencia* (Agency benefits)**		Overall	5.23 (0.86)	0.77 *N* = 51
		1. Mejor reputación	5.45 (0.81)	
		2. Mayor habilidad de afectar política publica	5.08 (1.19)	
		3. Incremento en el uso por otros de la experiencia o los servicios de la agencia	5.14 (1.08)	
***Relaciones de Poder en la Investigación* (Community power in research)**		Overall	6.16 (1.05)	0.93 *N* = 52
		1. Han aumentado su participación en el proceso de la investigación	5.81 (1.37)	
		2. Pueden hablar sobre el proyecto con grupos o en otras escenas, como en juntas comunitarias o políticas	6.31 (0.98)	
		3. Pueden aplicar los resultados de la investigación a practica y programas en la comunidad	6.21 (1.21)	
		4. Pueden expresar sus opiniones sobre la investigación en frente a investigadores	6.27 (1.09)	
		5. Tienen la capacidad o el poder de promover investigaciones que beneficiaran a la comunidad	6.19 (1.24)	
***Sostenibilidad del Proyecto* (Sustainability)**		Overall	6.21 (0.78)	0.45 *N* = 56
		1. Estoy comprometido(a) a mantener la relación comunitaria-académica sin fondos o con pocos fondos	6.50 (0.71)	
		2. Probablemente este proyecto continuará después de que se terminen los fondos	5.89 (1.41)	
		3. Nuestra colaboración cuidadosamente evalúa las oportunidades de financiamiento para estar seguros de que reflejen las necesidades de ambos colegas comunitarios y académicos	6.23 (1.16)	
***Futuros resultados (*Projected outcomes)**				
***Política* (Policy)**		Overall	4.76 (1.10)	0.87 *N* = 52
		1. Mejor coordinación entre agencias, investigadores, y grupos comunitarios	5.04 (1.12)	
		2. Cambios en el carácter de debates sobre temas importantes de salud en la comunidad	5.02 (1.01)	
		3. Resultados útiles para el desarrollo, en la comunidad, de prácticas comunitarias, programas, o políticas públicas	5.04 (1.05)	
		4. Cambios en política pública	3.96 (1.79)	
***Integracion de la comunidad en investigación* (Community integration in research)**		Overall	5.14 (1.07)	0.91 *N* = 55
		1. Mejor habilidad académica para integrar perspectivas comunitarias en el diseño y metodología de las investigaciones	5.15 (1.10)	
		2. Investigaciones con mejores conexiones a las necesidades comunitarias	5.13 (1.14)	
***Transformación social* (Social transformation)**		Overall	4.76 (1.22)	0.91 *N* = 50
		1. Fortalecimiento de la identidad cultural u orgullo	4.88 (1.22)	
		2. Impactos sociales amplios	4.52 (1.46)	
		3. Mejor ambiente general en la comunidad	27.88 (1.27)	
***Mejoria de Salud* (Health Improvement)**		Overall	5.14 (0.91)	0.94 *N* = 57
		1. ¿Cuánto cree usted que este proyecto mejorará la salud de la comunidad?	5.16 (0.96)	
		2. ¿Cuánto cree usted que este proyecto mejorara los comportamientos de salud en los miembros de la comunidad?	5.12 (0.93)	

Correlations between all subscales are reported in Table 3 across the four CBPR domains. Scales 1–3 represent the CBPR domain Context, and scales 4–11 represent Partnership Processes. Scales 12–15 correspond to Intervention and Research, and scales 16–23 represent Outcomes. Significant correlations were found between and within domains of measures. For scales representing the CBPR domains Context and Partnership Processes, the strongest positive correlations were between partnership capacity and leadership, *r* = 0.641, *p* < 0.01, and bridging differences and leadership, *r* = 0.609, *p* < 0.01. The strongest correlation between Context and Intervention and Research Action domains was between partnership capacity and synergy, *r* = 0.478, *p* < 0.01, and bridging differences and analyses and dissemination, *r* = 0.433, *p* < 0.01. For Context and Outcomes, the highest correlation was between partnership capacity and community power in research, *r = 0*.617¸ *p* < 0.01, and bridging differences and community integration *r* = 0.544, *p* < 0.01.

**Table 3.** Correlation matrix for Encuesta Comunitaria (Spanish Community Engagement Survey)

	1	2	3	4	5	6	7	8	9	10	11	12	13	14	15	16	17	18	19	20	21	22	23
**Context**																							
**Capacity**																							
1. Community history	–	0.572 **	0.458 **	0.486 **	0.468 **	0.263	0.271*	0.356 **	0.023	0.028	0.292 *	0.251	0.288*	0.054	0.353 **	0.232	0.389 **	0.312 *	0.118	0.250	0.400 **	0.313 *	0.157
2. Partnership capacity	0.572 **	–	0.803 **	0.415 **	0.404 **	0.254	0.232	0.641 **	0.265	0.279 *	0.445 **	0.309 *	0.267	0.211	0.478 **	0.384 **	0.425 **	0.617 **	0.319 *	0.379 **	0.503 **	0.461 **	0.444 **
3. Bridging differences	0.458 **	0.803 **	–	0.469 **	0.492 **	0.249	0.295 *	0.609 **	0.387 **	0.377 **	0.488 **	0.447 **	0.439 **	0.359 *	0.433 **	0.244	0.449 **	0.486 **	0.249	0.420 **	0.544 **	0.464 **	0.326 *
**Partnership Processes**																							
**Commitment to collective empowerment**																							
4. Partnering principles	0.486 **	0.415 **	0.469 **	–	0.661 **	0.380 **	0.601 **	0.648 **	0.392 **	0.381 **	0.295 *	0.525 **	0.456 **	0.429 **	0.705 **	0.180	0.333 *	0.455 **	0.125	0.424 **	0.607 **	0.490 **	0.364 **
5. Community fit	0.468 **	0.404 **	0.492 **	0.661 **	–	0.272 *	0.366 **	0.482 **	0.422 **	0.265	0.383 **	0.340 *	0.312 *	0.154	0.609 **	-0.011	0.282 *	0.192	0.078	0.399 **	0.627 **	0.443 **	0.471 **
6. Influence in the partnership	0.263	0.254	0.249	0.380 **	0.272 *	–	0.414 **	0.234	0.547 **	0.440 **	0.401 **	0.383 **	0.311 *	0.311 *	0.515 **	0.059	-0.112	0.242	-0.042	0.264	0.344 *	0.281	0.165
7. Collective reflection	0.271 *	0.232	0.295 *	0.601 **	0.366 **	0.414 **	–	0.345 **	0.169	0.265 *	0.119	0.307 *	0.250	0.303 *	0.502 **	0.286 *	0.136	0.339 *	0.212	0.357 **	0.271 *	0.305 *	0.278 *
**Relationships**																							
8. Leadership	0.356 **	0.641 **	0.609 **	0.648 **	0.482 **	0.234	0.345*	–	0.354 **	0.242	0.407**	0.477 **	0.448 **	0.462 **	0.496 **	0.498 **	0.514 **	0.628 **	0.419 **	0.473 **	0.618 **	0.571 **	0.458 **
9. Dialogue	0.023	0.265	0.387 **	0.392 **	0.422 **	0.547 **	0.169	0.354 **	–	0.720 **	0.513 **	0.295 *	0.222	0.296 *	0.506 **	0.013	0.027	0.385 **	0.026	0.361 *	0.467 **	0.346 *	0.345 *
10. Conflict resolution	0.028	0.279 *	0.377 **	0.381 **	0.265	0.440 **	0.265 *	0.242	0.720 **	–	0.404 **	0.306 *	0.210	0.378 **	0.405 **	0.068	0.142	0.376 **	0.037	0.495 **	0.417 **	0.417 **	0.276 *
11. Trust	0.292 *	0.445 **	0.488 **	0.295 *	0.383 **	0.401 **	0.119	0.407 **	0.513 **	0.404 **	–	0.183	0.245	0.169	0.345 **	0.129	0.074	0.309 *	0.253	0.126	0.250	0.233	0.156
**Intervention and Research**																							
**Community engagement in research actions**																							
12. Background and design	0.251	0.309 *	0.447 **	0.525 **	0.340 *	0.383 **	0.307 *	0.477 **	0.295 *	0.306 *	0.183	–	0.858 **	0.747 **	0.413 **	0.194	0.435 **	0.416 **	0.098	0.430 **	0.456 **	0.490 **	0.231
13. Analyses and dissemination	0.288 *	0.267	0.439 **	0.456 **	0.312 *	0.311 *	0.250	0.448 **	0.222	0.210	0.245	0.858 **	–	0.793 **	0.424 **	0.129	0.331 *	0.260	0.069	0.476 **	0.446 **	0.499 **	0.232
14. Community action	0.054	0.211	0.359 *	0.429 **	0.154	0.311 *	0.303 *	0.462 **	0.296 *	0.378 **	0.169	0.747 **	0.793 **	–	0.352 *	0.123	0.367 *	0.288 *	0.204	0.504 **	0.347 *	0.483 **	0.264
**Synergy**																							
15. Synergy	0.353 **	0.478 *	0.433 **	0.705 **	0.609 **	0.515 **	0.502 **	0.496 **	0.506 **	0.405 **	0.345 *	0.413 **	0.424 **	0.352 *	–	0.087	0.298 *	0.441 **	0.051	0.438 **	0.567 **	0.541 **	0.468 **
**Outcomes**																							
**Systems and capacity change**																							
16. Personal benefits	0.232	0.384 **	0.244	0.180	-0.011	0.059	0.286 *	0.498 **	0.013	0.068	0.129	0.194	0.129	0.123	0.087	–	0.449 **	0.567 **	0.263	0.411 **	0.307 *	0.482 **	0.274 *
17. Agency benefits	0.389 **	0.425 **	0.449 **	0.333 *	0.282 *	-0.112	0.136	0.514 **	0.027	0.142	0.074	0.435 **	0.331 *	0.367 *	0.298 *	0.449 **	–	0.512 **	0.340 *	0.479 **	0.407 **	0.609 **	0.322 *
18. Community power in research	0.312 *	0.617 **	0.486 **	0.455 **	0.192	0.242	0.339 *	0.628 **	0.385 **	0.376 **	0.309 *	0.416 **	0.260	0.288 *	0.441 **	0.567 **	0.512 **	–	0.440 **	0.352 *	0.593 **	0.473 **	0.389 **
19. Sustainability	0.118	0.319 *	0.249	0.125	0.078	-0.042	0.212	0.419 **	0.026	0.037	0.253	0.098	0.069	0.204	0.051	0.263	0.340 *	0.440 **	–	0.305 *	0.299 *	0.290 *	0.241
**Projected outcomes**																							
20. Policy	0.250	0.379 **	0.420 **	0.424 **	0.399 **	0.264	0.357 **	0.473 **	0.361 *	0.495 **	0.126	0.430 **	0.476 **	0.504 **	0.438 **	0.411 **	0.479 **	0.352 *	0.305 *	–	0.719 **	0.878 **	0.581 **
21. Commnity integration	0.400 **	0.503 **	0.544 **	0.607 **	0.627 **	0.344 *	0.271 *	0.618 **	0.467 **	0.417 **	0.250	0.456 **	0.446 **	0.347 *	0.567 **	0.307 *	0.407 **	0.593 **	0.299 *	0.719 **	–	0.720 **	0.508 **
22. Social transformation	0.313 *	0.461 **	0.464 **	0.490 **	0.443 **	0.281	0.305 *	0.571 **	0.346 *	0.417 **	0.233	0.490 **	0.499 **	0.483 **	0.541 **	0.482 **	0.609 **	0.473 **	0.290 *	0.878 **	0.720 **	–	0.683 **
23. Health improvements	0.157	0.444 **	0.326 *	0.364 **	0.471 **	0.165	0.278 *	0.458 **	0.345 *	0.276 *	0.156	0.231	0.232	0.264	0.468 **	0.274 *	0.322 *	0.389 *	0.241	0.581 **	0.508 **	0.683 **	–

* < 0.05, ** < 0.01. The bolded constructs on the left are the scales.

The strongest relationship between Partnership Processes and Intervention and Research Action was between partnering principles and synergy, *r* = 0.705 *p* < 0.01, and community fit and synergy, *r* = 0.609, *p* < 0.01. For Partnership Processes and Outcomes, the highest correlation was between leadership and community power in research, *r* = 0.628, *p* < 0.01, and community fit and community integration, *r* = 0.627, *p* < 0.01. The strongest relationship between the scales in the Intervention and Research and Outcomes domains was between synergy and community integration, *r* = 0.567, *p* < 0.01, and synergy and social transformation, *r* = 0.541, *p* < 0.01.

### Development of the shorter measure: *Fortaleciendo y Uniendo EsfueRzos Transdisciplinarios para Equidad de Salud* (FUERTES)

Given the length of the original measure and participant burden, additional expert Delphi consultation and psychometric work was performed to shorten the E^2^ CES from 93 items to 34 items to provide actionable data to partnerships more flexibly and practically. The goal was that an easy-to-use measure would allow partnerships to engage in annual evaluations and collective reflection about what is working well and what to strengthen in the coming year. Furthermore, a shorter measure enhances the uptake of the Spanish and English versions of the E^2^ CES in the dissemination and implementation phases of the broader E^2^ “Collective Reflection and Measurement Toolkit” (see tools on engageforequity.org).

Details on shortening the measure can be found elsewhere. Briefly, E^2^ Think Tank members and E^2^ community partners participated in a systematic Delphi process of categorizing E^2^ CES scales and individual items according to their importance for being included in the shorter measure (from “least important” to “most important: needs to be included”). Classical test theory and item response theory were then utilized to select items that allowed for the preservation of psychometric properties [26]. Each E^2^ CES item was evaluated and ranked in four areas: consistency within a scale or construct, convergent validity, information, and responsiveness to change, resulting in scales often reduced from 3 to 5 items to a single item. Additional input was received from E^2^ Think Tank members, E^2^ community partners, and respondents of the original E^2^ CES measure before finalizing the shorter version, the PHIRE survey.

#### Spanish translation and adaptation

To develop the short Spanish version, FUERTES, we performed a similar translation and adaptation process used during the development of the *Encuesta Comunitaria* (see Figure 1). Bilingual members of our team with origins from different parts of Latin America (e.g., Mexico, Cuba, Colombia, and Puerto Rico) with personal and professional experience working with diverse Spanish speakers across various research projects and settings in the U.S. (e.g., clinical, educational, and community) were involved. First, the original PHIRE survey was translated from English to Spanish by two independent CBPR bilingual researchers (JP and PRE). This translation was independently reviewed and further refined by a professional certified translator (DR). After the initial Spanish translation, these two researchers met regularly with additional bilingual CBPR researchers, including members of the national E^2^ Think Tank. They discussed each item while comparing it to the English version. During six months, members of the bilingual research team (*n* = 8) met every other week or monthly for 1.5 hours to compare, discuss discrepancies, and arrive at a consensus of items and response options.

To avoid confusion when responding to negative items in scales, we decided to reverse two negative items to the positive sense [27]. For example, under the Colaboraciones (Partnering) domain, the negative item, “Cuando tenemos conversaciones, con frecuencia nos malinterpretamos” was changed to “Nosotros nos escuchamos.” This resulted in similar changes in the English PHIRE version. For instance, the negative item “When we have conversations, we often misunderstand each other” was changed to “When we have conversations, we mostly understand each other.” These discussions also helped us find more understandable and equivalent response options. Under the Características (Characteristics) domain, we initially started with the response options: de ningún modo, a un modo pequeño, a un modo moderado, a un gran modo, and a un muy gran modo. Through several dialogs in our meetings, we recognized that it was difficult to distinguish these response options and decided to change them to avoid confusion and ensure better delineation among the options. As seen in Table 4, the final response options were nunca, casi nunca, en ocasiones, con frecuencia, and casi siempre. See Table 4 for the full items and response options.

**Table 4.** English and Spanish items in the FUERTES measures

Scale and items	Survey question (English/Spanish)	Response options
**Partnership capacity/*Capacidad de colaboración***	Does your partnership have any of the following capacities to achieve the project aims? 1. Community legitimacy and trust 2. Ability to bring people together for meetings/activities 3. Academic partners with the skills and experience needed to interact effectively with community partners 4. Community partners with the skills and experience needed to interact effectively with academic partners 5. Diverse partners, reflecting the community	Not at all To a small extent To a moderate extent To a great extent To a very great extent
	Esta colaboración, ¿tiene alguna de las siguientes características para lograr los objetivos del proyecto? 1. La comunidad confía en la colaboración 2. Habilidad de congregar a personas en reuniones/actividades 3. Colegas académicos con las habilidades y experiencia necesarias para interactuar efectivamente con los colegas/colaboradores de la comunidad 4. Colegas comunitarios con las habilidades y experiencia necesaria para interactuar efectivamente con los colegas académicos/de investigación 5. Colegas diversos que reflejan a la comunidad	Nunca Casi nunca En ocasiones Con frecuencia Casi siempre
**Partnering/*Colaboraciones***	How much do you agree or disagree that in this partnership: 6. We can rely on each other 7. We have consensus about the priorities of our partnership 8. Our leadership encourages active participation of all partners in decision making 9. When we have conversations, we often understand each other 10. Even when we don’t have total agreement, we reach a kind of consensus that we all accept	Strongly disagree Disagree Neither agree nor disagree Agree Strongly agree
	¿Qué tan de acuerdo o en desacuerdo está con que en esta colaboración: 6. Podemos confiar uno en el otro 7. Estamos de acuerdo con las prioridades en nuestra colaboración 8. Nuestro liderazgo promueve la participación activa de todos los colegas en el proceso de tomar decisiones 9. Nosotros nos escuchamos 10. Aun cuando no estamos completamente de acuerdo, logramos un consenso que todos podemos aceptar	Totalmente en desacuerdo En desacuerdo Ni de acuerdo ni en desacuerdo De acuerdo Totalmente de acuerdo
**Collective Empowerment/*Empoderamiento colectivo***	11. We all have influence over decisions that our partnership makes 12. We evaluate together what we’ve done well and how we can improve our collaboration 13. We partner equitably while promoting social and health equity 14. Our project builds on resources and strengths in the community 15. Our partnership honors community contributions to knowledge	Not at all To a small extent To a moderate extent To a great extent To a very great extent
	11. Todos tenemos influencia sobre las decisiones que nuestra colaboración toma 12. Juntos evaluamos lo que hemos hecho bien y cómo podemos mejorar nuestra colaboración. 13. Colaboramos de una manera equitativa mientras promovemos equidad social y de la salud 14. Nuestro proyecto se basa en los recursos y fortalezas de la comunidad 15. Nuestra colaboración honra las contribuciones del conocimiento se la comunidad	Nunca Casi nunca En ocasiones Con frecuencia Casi siempre
**Governance and Resource Sharing/ *Gobernanza e intercambio de recursos***	This section of the PHIRE survey tool asks about any structures your project has in place to ensure ongoing accountability to community needs and fairness in the sharing of project resources. The term community-based oversight body is used in this section of the survey tool to refer to any group, board, or governing body that has some autonomy from the core research partnership and provides continuing, community-based oversight of the research process. Examples may include a community advisory board, the leadership of a community-based organization, or a local or tribal government. Please feel free to answer “I don’t know” to any question you are unsure about. 16. There is a community-based oversight body that ensures that this project benefits the community 17. Community and academic partners decide together how to share the project’s financial resources 18. Resources are shared fairly in this project	Not at all To a small extent To a moderate extent To a great extent To a very great extent I don’t know
	Esta sección de la encuesta FUERTES pregunta sobre como se asegura que la colaboración sea justa dentro de la comunidad. Para asegurar que sea justa, un **comité de supervisión comunitaria** como una junta asesora comunitaria, el liderazgo de la organización comunitaria o un gobierno local o tribu asegura la responsabilidad continua en el proceso de investigación. Puede responder “No sé” a cualquier pregunta donde no esté seguro. 16. Existe un comité de supervisión comunitario que asegura que este proyecto beneficia a la comunidad 17. Los colegas comunitarios y académicos deciden juntos cómo compartir los recursos financieros del proyecto 18. Los recursos se comparten de manera justa en este proyecto	Nunca Casi nunca En ocasiones Con frecuencia Casi siempre No sé
**Community Advisory Board (CAB) Functions/*Funciones de la Junta Asesora Comunitaria (JAC)***	If you have a CAB or other oversight board, how much have community partners been involved in the following steps to develop their board? 19. Designing and implementing the CAB 20. Interpreting CAB discussions 21. Disseminating useful information from the CAB for community action and benefit 22. Informing relevant policy makers about CAB information	Not at all involved Slightly involved Moderately involved Very involved Extremely involved
	Si tiene un JAC u otro comité de supervisión, ¿cuánto han participado los colegas comunitarios en los siguientes pasos para desarollar su mesa directiva? 19. Diseño e implementación de la JAC 20. Interpretación de los debates de la JAC 21. Compartir información útil de la JAC para la acción y el beneficio de la comunidad 22. Comunicar a los legisladores y políticos pertinentes sobre la información de la JAC	Nunca Casi nunca En ocasiones Con frecuencia Casi siempre
**Community engaged in research action/*Participación comunitaria en acción de investigación***	How much have community partners been involved in the following research steps? For steps that have not yet happened, how much will community members be involved? 23. Designing and implementing the intervention and/or study 24. Interpreting study findings 25. Disseminating useful findings for community action and benefit 26. Informing relevant policy makers about findings	Not at all involved Slightly involved Moderately involved Very involved Extremely involved
	¿Qué tanto los colegas comunitarios han participado en las siguientes fases de la investigación? Para las fases que aún no se han realizado, ¿qué tanto participarán los colegas comunitarios? 23. Diseño e implementación del proyecto 24. Interpretación de los resultados 25. Compartir los resultados del proyecto para promover acciones y beneficios para la comunidad 26. Comunicar los resultados del proyecto a los políticos y legisladores pertinentes	Nunca Casi nunca En ocasiones Con frecuencia Casi siempre
**Partnership synergy/*Sinergía de la Colaboración***	27. We work together effectively as a community/academic partnership.	Strongly disagree Disagree Neither agree nor disagree Agree Strongly agree
	27. Trabajamos juntos de manera efectiva en una colaboración comunitaria/académica	Totalmente en desacuerdo En desacuerdo Ni de acuerdo ni en desacuerdo De acuerdo Totalmente de acuerdo
**Benefits/*Beneficios***	How much do you agree or disagree that as a result of this project: 28. Academic partners have come to value community knowledge more and more 29. All partners experience personal benefits such as enhanced reputation or increased use of skills and expertise by others 30. Community members have increased power to promote research that will benefit the community 31. Organizations involved in this partnership have increased power to improve community health	Strongly disagree Disagree Neither agree nor disagree Agree Strongly agree
	¿Qué tan de acuerdo o desacuerdo está con los siguientes beneficios del proyecto: 28. Los colegas académicos han llegado a valorar cada vez más el conocimiento comunitario 29. Todos los colegas obtienen beneficios personales, tales como tener una mejor reputación o un aumento del uso de sus habilidades y experiencia por otros. 30. Los miembros de la comunidad tienen mayor poder para promover investigaciones que beneficiarán a la comunidad 31. Las organizaciones que participan en esta colaboración han aumentado su poder para mejorar la salud de la comunidad	Totalmente en desacuerdo En desacuerdo Ni de acuerdo ni en desacuerdo De acuerdo Totalmente de acuerdo
**Future Outcomes/*Resultados Futuros***	How much will this project produce: 32. Useful findings for the development of practices, programs, or policies 33. Research better linked to community needs 34. Improved community health	Not at all To a small extent To a moderate extent To a great extent To a very great extent
	¿Cuánto producirá este proyecto: 32. Resultados que serán usados para el desarrollo de prácticas, programas o políticas públicas 33. Mejor investigaciones vinculadas a las necesidades de la comunidad 34. Mejoría de la salud comunitaria	Nunca Casi nunca En ocasiones Con frecuencia Casi siempre

## Discussion

The present study contributes to the development of validated Spanish and pragmatic measures for CEnR scholarship that are culturally adapted for use in the US and Latin America. The two instruments, *Encuesta Comunitaria* and its shorter version, FUERTES, can meet the needs for evaluating or assessing academic-community partnering practices and outcomes involving Spanish-speaking partners and communities. Reviewing response means of aggregated data for each scale enables partners to reflect together on which practices their partnership is implementing well; how these compare with national best practices; and ultimately, what practices they could strengthen in subsequent years. Stratification of data by stakeholder groups may enable a deeper dialog into areas of alignment or misalignment. The goal of these instruments is for dialog and action, with the opportunity for annual implementation and reflection/action, not simply to capture one point in time.

Reflexive practices [28] – or the continuous dialog, reflection, and action around research and partnership practices – are among the recognized empirically-based best practices of CEnR and CBPR [1,29]. Collective reflexive practices have been shown to contribute to capacity building, enhanced adherence to CBPR principles, equitable power-sharing, and partnership sustainability [1]. This reflection, facilitated by the use of these measurement instruments can support partnerships in deeper engagement, reflecting on their partnership goals, and their progress towards achieving intended outcomes, including those of health equity and social justice. Using the *Encuesta Comunitaria* instrument can support partners to assess current performance and progress over time with constructs that correspond to the CBPR and NAM models [5,14]. The shorter pragmatic instrument, FUERTES, may more easily facilitate annual collective reflection assessments of partnership practices while limiting burden to partners during completion.

### Future directions

Future studies using the *Encuesta Comunitaria* could assess which subdomains best contribute to specific outcomes prioritized by the partnerships, including policy changes, improved health, access to new or enhanced resources, or sustainability of such changes and the partnership itself. Moreover, future studies are also needed to further validate and refine both instruments with consideration of the heterogeneity of the Latino/a/x population. For example, in New Mexico, a state-wide CBPR training, involving the Department of Health, the University of New Mexico, New Mexico State University, and local communities, has produced a module that incorporates PHIRE and FUERTES instruments for diverse collaborative projects to engage in collective evaluation. Discussion is also underway for application with the new NIH-funded ComPASS projects. As additional partnerships use the two Spanish tools, we will continue to refine them based on psychometrics and feedback.

Finally, the UNM CPR is producing a web app that enables easy implementation with partners, data analysis, and production of a partnership data report for collective reflection. The web app will allow Principal Investigators or project coordinators to input the names and emails of project partners, including academic faculty, staff and students, community researchers, community advisory board members, or other partners. An Excel spreadsheet will provide calculated means for each scale, with the means then inputted into a colorful partnership data report that includes questions to generate partner dialog on their strengths and future strategic actions.

### Limitations

The proposed measures have some limitations to consider. There is an inherent challenge in creating universal Spanish instruments due to variations of the language by country, regions, and other contextual factors. While our translation and adaptation process included partners representing different countries and linguistic backgrounds, we encourage users to review with their partners, before use, to ensure the measures address their local context and language needs and that they address concepts or principles that their partnership most cares about. In addition to the translation and back-translation process, an adaptation of the instrument is a key step to ensure a high-quality translation and that the final instrument is one that will be understood by the intended audiences [30]. Due to the limited number of Spanish-speaking US partnerships that completed the measures during the E^2^ CES testing in 2020, we were unable to meaningfully analyze their data. However, as previously stated, as additional CEnR and CBPR partnerships use both measures, we will continue to adapt and optimize them.

## Conclusion

The *Encuesta Comunitaria* and the FUERTES measures offer CEnR and CBPR practitioners and partnerships two validated tools for assessing research, practices, and outcomes of their work. They contribute to ongoing national and international efforts, as recently highlighted by the National Academies of Medicine, to develop, test, and disseminate measures of meaningful community engagement and CBPR. Moreover, our development of a short tool, FUERTES, meets the needs of partnerships looking for pragmatic tools to incorporate evaluation into their efforts without adding undue burden to their partners. Updating these measures through reflexive practices will further strengthen CEnR and CBPR involving Latinx communities within the US, Latin America, and globally.
